# Advances in metabolic reprogramming of renal tubular epithelial cells in sepsis-associated acute kidney injury

**DOI:** 10.3389/fphys.2024.1329644

**Published:** 2024-01-19

**Authors:** Tiantian Wang, Ying Huang, Xiaobei Zhang, Yi Zhang, Xiangcheng Zhang

**Affiliations:** Department of Critical Care Medicine, The Affiliated Huaian No 1 People’s Hospital of Nanjing Medical University, Huaian, Jiangsu, China

**Keywords:** sepsis-associated acute kidney injury, metabolic reprogramming, renal tubular epithelial cells, oxidative phosphorylation, glycolysis

## Abstract

Sepsis-associated acute kidney injury presents as a critical condition characterized by prolonged hospital stays, elevated mortality rates, and an increased likelihood of transition to chronic kidney disease. Sepsis-associated acute kidney injury suppresses fatty acid oxidation and oxidative phosphorylation in the mitochondria of renal tubular epithelial cells, thus favoring a metabolic shift towards glycolysis for energy production. This shift acts as a protective mechanism for the kidneys. However, an extended reliance on glycolysis may contribute to tubular atrophy, fibrosis, and subsequent chronic kidney disease progression. Metabolic reprogramming interventions have emerged as prospective strategies to counteract sepsis-associated acute kidney injury by restoring normal metabolic function, offering potential therapeutic and preventive modalities. This review delves into the metabolic alterations of tubular epithelial cells associated with sepsis-associated acute kidney injury, stressing the importance of metabolic reprogramming for the immune response and the urgency of metabolic normalization. We present various intervention targets that could facilitate the recovery of oxidative phosphorylation-centric metabolism. These novel insights and strategies aim to transform the clinical prevention and treatment landscape of sepsis-associated acute kidney injury, with a focus on metabolic mechanisms. This investigation could provide valuable insights for clinicians aiming to enhance patient outcomes in the context of sepsis-associated acute kidney injury.

## Background

Sepsis-associated acute kidney injury (SA-AKI) is characterized by the presence of both consensus sepsis criteria (as defined by Sepsis-3 recommendations) and acute kidney injury (AKI) criteria [as defined by Kidney Disease: Improving Global Outcomes (KDIGO) recommendations] when AKI occurs within 7 days from diagnosis of sepsis (Kidney Int Suppl, 2011; Singer et al., 2016; Zarbock et al., 2023). The prevalence of SA-AKI is associated with increased mortality rates, lengthy stays in intensive care units, extended hospitalizations, greater long-term impairments, and reduced quality of life (Schuler et al., 2018; Poston and Koyner, 2019; Stanski et al., 2020; Zarbock et al., 2023).

While the precise pathophysiological mechanisms underlying SA-AKI remain incompletely delineated, contributing factors are acknowledged to encompass systemic and renal inflammation, complement activation, dysregulation of the renin-angiotensin-aldosterone system, mitochondrial dysfunction, metabolic reprogramming, and vascular anomalies that span both microcirculatory dysfunction and macrocirculatory alteration (Zarbock et al., 2023).

During the early phase of sepsis, renal tubular epithelial cells (TECs) switch from oxidative phosphorylation (OXPHOS), a highly efficient energy production pathway, to aerobic glycolysis, which is substantially less efficient (Gómez et al., 2017). This metabolic shift is thought to be an essential protective response to infection. This review highlights various therapeutic targets that have been identified to modulate this metabolic adaptation, potentially pioneering new clinical approaches for managing SA-AKI.

## Unveiling TECs’ metabolic machinery: exploring normal metabolism

The kidney plays a pivotal role in reabsorbing glucose, ions, and nutrients via ion channels and transporters located in the proximal tubules. The energy requisite for this selective reabsorption is primarily supplied by the kidney’s mitochondria, which produce ATP to fuel the Na^+^-K^+^ ATPase pump, a process driven by the ionic gradient this pump establishes. The study suggests that this reabsorption process is responsible for up to 70% of the kidney’s total energy consumption (Bhargava and Schnellmann, 2017).

**FIGURE 1:**
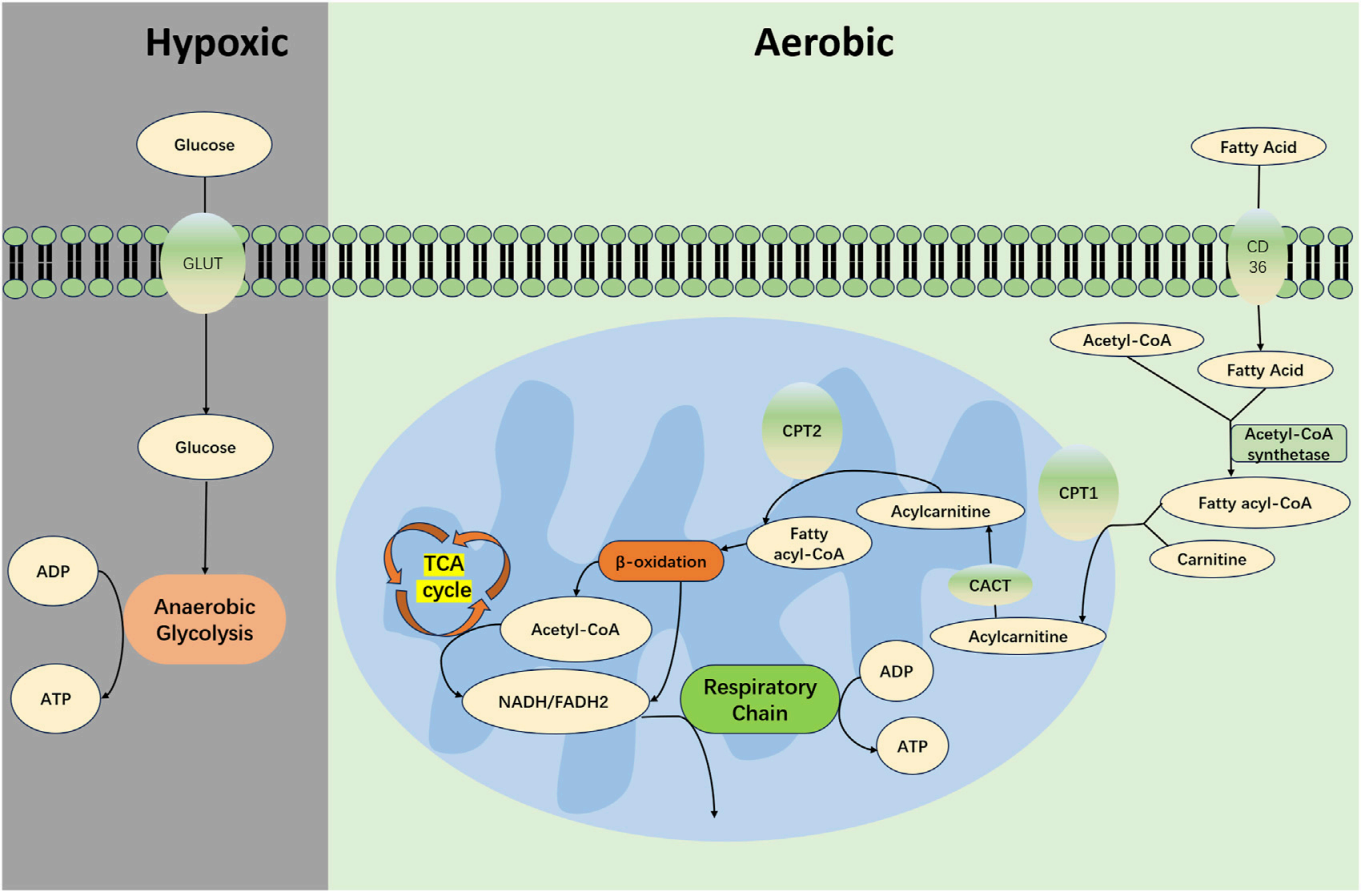
Metabolism in Normal Renal TECs. In the proximal tubules of the kidney, long-chain fatty acids are absorbed via transport proteins such as CD36 and fatty acid binding protein (FABP). These fatty acids are catalyzed by acyl-CoA synthetase on the outer mitochondrial membrane to form acyl-CoA in conjunction with coenzyme A (CoA). Acyl-CoA is then shuttled through a channel into the mitochondrial intermembrane space where it encounters carnitine palmitoyltransferase 1 (CPT1) that is embedded in the membrane. Under the catalysis of CPT1, acyl-CoA is conjugated with carnitine to form acylcarnitine. This acylcarnitine is then transported across the mitochondrial inner membrane by carnitine-acylcarnitine translocase (CACT). Subsequently, acylcarnitine is converted back to acyl-CoA by carnitine acyltransferase 2 (CPT2), which is associated with the mitochondrial inner membrane. Acyl-CoA then undergoes a series of reactions including dehydrogenation, hydration, dehydrogenation, and thiolytic cleavage, constituting the β-oxidation process. This leads to the production of acetyl-CoA, while concomitantly generating FADH2 and NADH. The acetyl-CoA enters the TCA cycle, resulting in additional molecules of FADH2 and NADH. These electron carriers—FADH2 and NADH—from both the β-oxidation and the TCA cycle partake in the respiratory chain, transferring electrons to oxygen atoms; this electron transfer event is coupled with the phosphorylation of ADP to create ATP. Under conditions of oxygen deprivation, cells primarily generate energy through anaerobic glycolysis.CD36, cluster of differentiation 36; TEC, Tubular Epithelial Cell; CPT-1, carnitine O-palmitoyltransferase 1; CACT, carnitine-acylcarnitine translocase; CPT-2, carnitine O-palmitoyltransferase 2; TCA, tricarboxylic acid cycle; FADH2, reduced flavin adenine dinucleotide; NADH, reduced nicotinamide adenine dinucleotide; ATP, adenosine triphosphate; ADP, adenosine diphosphate.

To fulfill these high energy requirements, renal TECs mainly depend on fatty acid oxidation (FAO) and OXPHOS for efficient energy production, as depicted in Figure 1 (Han et al., 2017; Harzandi et al., 2021). Specifically, TECs uptake long-chain fatty acids via transport proteins and subsequently facilitate their oxidation within the mitochondria (Yang et al., 2017). This process yields acetyl-CoA, reduced flavin adenine dinucleotide (FADH2), and reduced nicotinamide adenine dinucleotide (NADH) (Houten et al., 2016). Acetyl-CoA is further channeled into the tricarboxylic acid (TCA) cycle, creating additional FADH2 and NADH, which then feed into the electron transport chain to ultimately generate ATP (Gao and Chen, 2022). Given that the FAO-driven OXPHOS pathway can yield between 106 and 129 molecules of ATP per cycle, it is recognized as one of the most efficient pathways for cellular energy production (Jang et al., 2020).

Moreover, with renal TECs representing primary sites for FAO and OXPHOS, it is noteworthy that the kidneys contain the second-highest concentration of mitochondria in the body, following only the myocardium (Pagliarini et al., 2008). This substantial mitochondrial endowment is essential in meeting the rigorous energy demands of kidney function.

## Building resilience: exploring TECs’ resistance and tolerance mechanisms

Upon pathogen invasion, a host organism activates two cardinal defense strategies to combat the infection: resistance and tolerance (Schneider and Ayres, 2008).

Host resistance reduces the pathogen burden by blocking the entry of pathogens or eliminating them (Schneider and Ayres, 2008). The immune system is pivotal for resistance. While the innate immune system often effectively clears pathogens, it can sometimes be overwhelmed, leading to harmful host reactions. In sepsis, the immune response may become dysregulated, manifesting as excessive inflammation or suppression, both of which are harmful (van der Poll et al., 2017). Sepsis may trigger both inflammation and immune suppression sequentially or simultaneously (Liu D. et al., 2022). Upon invasion, immune cells, particularly renal TECs expressing Toll-like receptors (TLRs), identify pathogen-associated (PAMPs) and damage-associated molecular patterns (DAMPs). This interaction activates immune cascades (Jang and Rabb, 2015). Specifically, TLR2 and TLR4 initiate upregulation of specific genes to produce inflammatory cytokines (e.g., TNF-α, MCP-1) through NF-κB activation, leading to further signaling events (Naito et al., 2008; Fry, 2012). The cytokines produced promote the inflammatory response. TECs also sense PAMPs and DAMPs through TLR-4 on their basolateral membranes in the peritubular capillaries (Peerapornratana et al., 2019). This detection and subsequent activation of TECs’ innate immunity can cause oxidative stress, reactive oxygen species (ROS) production, and mitochondrial dysfunction, contributing to TEC injury (Kalakeche et al., 2011; Fry, 2012; Dellepiane et al., 2016). Hence, resistance during inflammation involves trade-offs and can result in immunopathology, compromising the host’s health (Toro et al., 2021).

Alongside resistance, the body also employs tolerance as a defense mechanism to minimize infection-induced tissue damage through multiple pathways (Medzhitov et al., 2012). The concept of tolerance, first introduced by Schafer (Schafer, 1971) and later expanded on by Schneider and Ayres (Schneider and Ayres, 2008) in 2018, applies to both animals and humans. Ganeshan, Nikkanen (Ganeshan et al., 2019) showed that in a mouse model of lipopolysaccharide (LPS)-induced sepsis, hepatic metabolism shifted from fatty acid oxidation toward acetyl-CoA and amino acid catabolism, resulting in a hypometabolic state that enables tissue tolerance and protection against bacterial infections. We hypothesize that sepsis-induced AKI may lead to metabolic reprogramming in the host, enhancing the state of tolerance to counteract damage not just from pathogens, but also from the host’s immune response.

## Mitochondrial dysfunction in SA-AKI: implications for TECs’ energy metabolism

Mitochondria, the sites of FAO and OXPHOS, are integral to the energy metabolism of TECs. In the context of SA-AKI, metabolic reprogramming is intimately associated with mitochondrial dysfunction. Research has demonstrated increases in mitochondrial DNA (mtDNA) oxidation and damage, alongside reductions in mitochondrial abundance in the kidneys of SA-AKI patients (van der Slikke et al., 2021). Mitochondrial dysfunction in the setting of sepsis precipitates ATP depletion and an upsurge in reactive oxygen species (ROS), both of which are contributory factors to cellular instability and subsequent renal organ dysfunction (Brealey et al., 2002). Yet, the exact molecular pathways through which mitochondria contribute to the pathogenesis of SA-AKI remain to be fully elucidated.

Mitochondrial damage within TECs during SA-AKI manifests as diminished mitochondrial mass, fragmentation, cristae disruption, and diverse extents of mitochondrial swelling (Parikh et al., 2015). Additionally, the challenges posed by sepsis extend to compromising the vital quality control mechanisms of mitochondria such as fission/fusion dynamics, biogenesis, and mitophagy—all of which are essential in preserving mitochondrial integrity and function.

For instance, dysfunctional mitochondria can initiate fission, resulting in two daughter mitochondria which may then fuse with healthy counterparts, aiming to ameliorate the damage (Sun et al., 2019). Nonetheless, this intricate process of mitochondrial fission and fusion becomes compromised during septic acute kidney injury (Liu et al., 2019).

Mitochondrial biogenesis, the process that elevates mitochondrial mass and enhances glycolytic and OXPHOS enzyme levels, ultimately increases mitochondrial energy production capabilities (Sun et al., 2019). Insufficient or delayed induction of mitochondrial biogenesis during critical illness magnifies the susceptibility to oxidative and nitrosative mitochondrial damage, impairs the clearance of various cellular organelles, and contributes to a net reduction in mitochondrial content, culminating in cellular energy deficits. Conversely, early initiation of mitochondrial biogenesis and prompt response to oxidative stress are associated with higher survival rates in patients with critical illnesses (Carré et al., 2010). Promoting biogenesis through diverse strategies in septic mouse models has proven effective in preserving renal function and improving survival outcomes (Jin et al., 2020; Li et al., 2023). Furthermore, *in vitro* analyses demonstrate that the NRF2 pathway enhances mitophagy in renal cells and tubules, stimulates mitochondrial biogenesis under septic conditions, increases the abundance of functional mitochondria, and promotes a more robust mitochondrial network, which collectively mitigate inflammation, oxidative stress, and apoptosis (Chen et al., 2022). These findings collectively underscore the significance of mitochondrial biogenesis in the recovery process of TECs post-injury.

Mitochondrial autophagy, otherwise designated as mitophagy, serves as an essential process for the selective identification, encapsulation, and breakdown of defective mitochondria, which is vital in the context of SA-AKI (Parikh et al., 2015). Therapeutic strategies that enhance mitophagy, such as the administration of melatonin which augments mitochondrial autophagy by enabling SIRT3-dependent deacetylation of transcription factor A (TFAM), have shown promise in mitigating the effects of SA-AKI (Deng et al., 2023).

## Metabolic shifts and reprogramming: unraveling changes and tolerance mechanisms in TECs during SA-AKI

**FIGURE 2:**
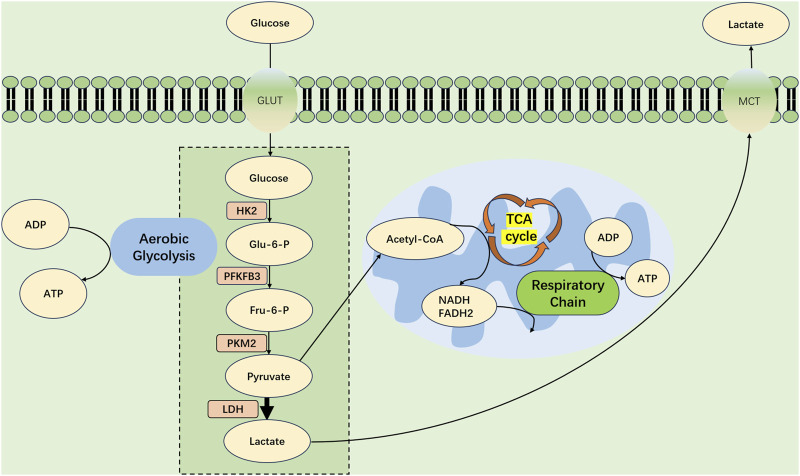
Metabolic Shift in Tubular Epithelial Cells During Septic Acute Kidney Injury. At the advent of SA-AKI, renal tubular epithelial cells (TECs) experience a profound metabolic transformation, migrating from OXPHOS toward a predominance of aerobic glycolysis. Within this metabolic realignment, the bulk of pyruvate forged by glycolytic processes eschews mitochondrial entry, opting instead for conversion into lactate—a process catalyzed by lactate dehydrogenase (LDH). This strategic metabolic adaptation is key, supporting an increased production of ATP through glycolysis to meet the amplified energy requirements imposed by the septic challenge. GLUT, glucose transporter protein; PFKFB3, fructose-2,6-bisphosphatase 3; HK2, hexokinase 2; PKM2, pyruvate kinase M2; Glu-6-P glucose 6-phosphate; Fru6-P Fructose 6-phosphate; LDH, Lactate Dehydrogenase; MCT, Monocarboxylate TransporterCD36, cluster of differentiation 36; TCA, tricarboxylic acid cycle; FADH2, reduced flavin adenine dinucleotide; NADH, reduced nicotinamide adenine dinucleotide; ATP, adenosine triphosphate; ADP, adenosine diphosphate.

After sepsis-induced AKI, renal TECs experience metabolic shifts, transitioning from FAO-driven OXPHOS to a glycolytic state, as depicted in Figure 2.

Researchers, utilizing a mouse model of cecal ligation and puncture (CLP)-induced sepsis, noted diminished renal fatty acid oxidase expression and a pronounced upregulation of glycolytic enzymes (Li et al., 2020).

Moreover, a dedicated metabolic and osmolyte study revealed significant post-CLP alterations within renal TECs; renal biopsies, obtained 8 hours after the procedure, demonstrated elevated glycolytic intermediates and reduced tricarboxylic acid (TCA) cycle intermediates (Waltz et al., 2016). Comparable metabolic shifts were observed in atrophic TECs associated with polycystic kidney disease and ischemia-reperfusion injury, processes mediated by activation of the Akt/phosphatidylinositol 3-kinase/mammalian target of rapamycin complex 1 (Akt/PI3K/mTORC1) signaling pathway (Lan et al., 2016; Podrini et al., 2020).

Notably, mTORC1 activation serves to stabilize the transcription factor hypoxia-inducible factor 1α (HIF-1α), thereby steering cellular metabolism toward aerobic glycolysis. Given these insights, it is plausible that TECs adapt their metabolism through similar mechanisms to other cell types under septic conditions.

This metabolic shift represents a protective, adaptive response that allows for enhanced infection tolerance, reduced cellular damage, and maintained viability (Liu J. et al., 2022). In their pivotal study, Katherine et al. discovered that glucose administration to malaria-infected mice bolstered glycolysis, leading to increased disease tolerance and enhanced survival rates (Cumnock et al., 2018).

During the early stages of LPS-induced AKI, the metabolic switch to glycolysis proves integral for establishing trained immunity (Cheng et al., 2014). Specifically, in LPS-induced SA-AKI mouse models, hexokinase (HK) activity, critical to the glycolytic pathway, markedly increased.

This HK surge facilitates activation of the pentose phosphate pathway (PPP), essential for maintaining glutathione (GSH) in a reduced state and upholding renal antioxidant defense mechanisms (Smith et al., 2014). In addition, aerobic glycolysis provides both sufficient energy and vital structural components, such as fatty acids, amino acids, and nucleotides, needed for cellular functions including mitosis (Vander Heiden et al., 2009).

## Restoring OXPHOS: rebalancing TECs’ metabolism

Initially, the organism’s metabolic shift toward glycolysis at the onset of sepsis may enhance tolerance and facilitate cellular survival; however, prolonged dependence on this pathway is ultimately deleterious. It becomes imperative for metabolism to transition back to OXPHOS. This necessity arises from several key findings.

First, upon SA-AKI onset, metabolic reprogramming in TECs leads to decreased utilization of free fatty acids and subsequent lipid accumulation (Jang et al., 2020). Such accumulation contributes to renal tubular fibrosis and glomerulosclerosis development (Gai et al., 2019). A recent investigation demonstrated that stimulating uncoupling protein 1 (UCP1) lessens lipid buildup, thereby inhibiting AKI progression through the AMPK/ULK1/autophagy pathway (Xiong et al., 2021).

Moreover, evidence suggests that timely metabolic restoration to OXPHOS and FAO can effectively rescue renal tubular ion transport and renal function (Toro et al., 2021). Activation of the farnesol X receptor (FXR) promotes FAO-related gene expression in TECs; this activation mitigates the effects of cisplatin-induced AKI and reduces lipid accumulation, protecting against FAO-mediated renal damage (Xu et al., 2022). These findings accentuate the therapeutic potential of targeting FXR and enhancing FAO in AKI treatment.

Furthermore, continuous aerobic glycolysis in renal TECs is linked to persistent local inflammation and compounded injury, reminiscent of the metabolic activity observed in immune cells (Toro et al., 2021; Zhu et al., 2022). Pyruvate kinase M2 (PKM2) plays a vital role in glycolysis’ final rate-limiting step, while high mobility group box 1 (HMGB1) acts as a powerful cytokine in the sepsis context. Elevated PKM2 levels have been shown to prompt HMGB1 release from macrophages, leading to inflammation. Remarkably, using a PKM2 inhibitor like picrotoxin can diminish HMGB1 levels, offering protection from sepsis (Yang et al., 2014).

Prolonged glycolysis during AKI also adversely influences renal tubular regeneration, progressing to tubular atrophy, fibrosis, and potential chronic kidney disease (CKD). Interventions enhancing FAO, activating PGC-1α with ZLN-005, or inhibiting glycolysis with 2-DG, a hexokinase 2 inhibitor, have proven to forestall the transition from AKI to CKD by preventing pericyte-to-myofibroblast transformation (Lan et al., 2016; Li et al., 2018; Xu et al., 2023).

While these findings illuminate the critical role of metabolic reprogramming in the prognosis of AKI to CKD, research on the appropriate timing for metabolic recovery from glycolysis to OXPHOS remains limited.

## Targeting treatment prospects: identifying potential therapeutic avenues

**FIGURE 3:**
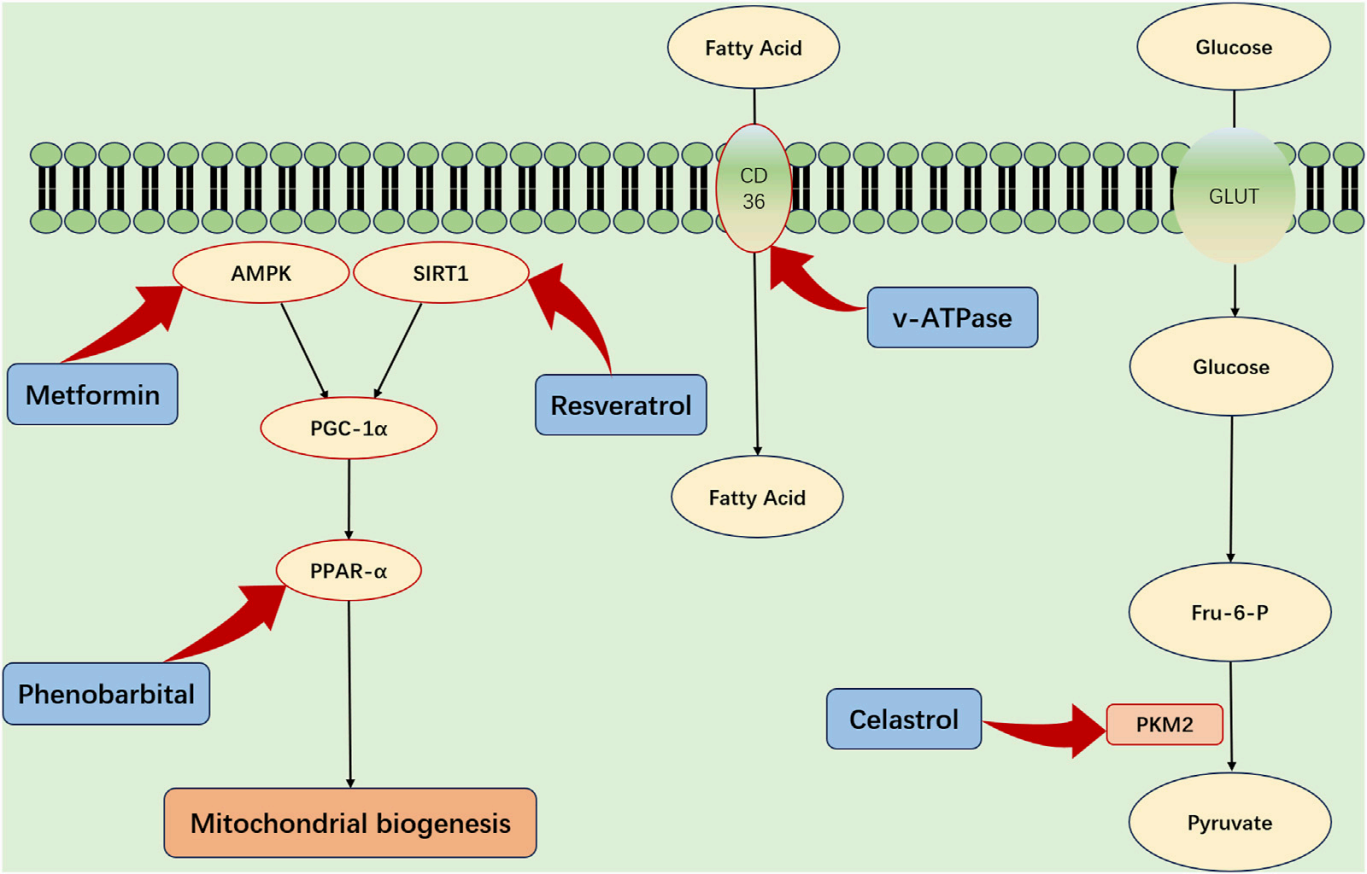
Strategic Metabolic Interventions for Renal Protection in SA-AKI. The refined activation of critical metabolic influencers—specifically PGC-1α, AMPK, SIRT1, and PPAR-α—and the enhancement of fatty acid absorption through CD36 mediation offer viable strategies for defending renal integrity amidst sepsis-associated acute kidney injury (SA-AKI). Additionally, strategically modulating PKM2, a key enzyme in the process of aerobic glycolysis, could provide a supplementary pathway to diminish the metabolic imbalances that are hallmark features of SA-AKI. AMPK, adenosine monophosphate kinase; PGC-1α, peroxisome proliferator-activated receptor gamma; Sirt1, sirtuin1; PPAR-α, peroxisome proliferator-activated receptor-α; TFAM, transcription factor A; GLUT, glucose transporter protein; PKM2, pyruvate kinase M2; Glu-6-P glucose 6-phosphate; Fru6-P Fructose 6-phosphate.

The metabolic functions of the kidneys are pivotal in the progression of SA-AKI. Accordingly, targeting renal energy metabolism emerges as a pivotal strategy for the prevention and treatment of SA-AKI (Figure 3). FAO and OXPHOS are the primary sources of renal energy, and the role of peroxisome proliferator-activated receptor gamma coactivator-1 alpha (PGC-1α) in regulating mitochondrial biogenesis is crucial. PGC-1α, which is principally expressed in the renal proximal tubular cells, becomes activated by AMPK phosphorylation and sirtuin1 (SIRT1) deacetylation, enhancing its regulatory functions (Fontecha-Barriuso et al., 2020).

During SA-AKI, a notable decline in PGC-1α expression impairs energy production, underscoring the significance of PGC-1α (Tran et al., 2011; Smith et al., 2015). Therefore, enhancing PGC-1α levels—possibly through the administration of recombinant human erythropoietin (rhEPO) or by pharmacologically stimulating the AMPK pathway—may help mitigate the detrimental effects of sepsis (Cantó and Auwerx, 2009; Jin et al., 2020; Li et al., 2023).

In addition, Peroxisome proliferator-activated receptor-α (PPAR-α) and CD36 are key modulators of renal metabolism. PPAR-α enhances FAO enzyme expression and aids fatty acid uptake via CD36 (Iwaki et al., 2019). Preclinical models indicate that PPAR-α agonists, such as fenofibrate, could offer protective benefits for renal health (Kaur et al., 2021). CD36 plays a critical role in allowing proximal tubular cells to obtain fatty acids attached to albumin from the bloodstream, which is essential for maintaining adequate energy levels (Yang et al., 2017). Targeted interventions aimed at CD36 might influence renal metabolism, though additional investigations are warranted to confirm this premise.

Additionally, targeting glycolysis in TECs may be considered a promising therapeutic strategy. PKM2, an enzyme crucial to the glycolytic pathway, and its inhibitors, notably celastrol and shikonin, are proposed to attenuate renal injury associated with SA-AKI (Wu et al., 2021; Luo et al., 2022).

In conclusion, manipulating metabolic pathways within the kidneys opens avenues for novel SA-AKI treatments. The strategic activation of PGC-1α pathways, stimulating the PPAR-α axis, and curtailing PKM2 activity present viable options for renal recovery. However, although these preliminary findings are hopeful, they require further exploration and verification through rigorous research.

## Discussion

Metabolic reprogramming may offer renal protection by enabling a tolerance mechanism during the initial stages of SA-AKI. However, a prolonged dependence on aerobic glycolysis may result in adverse outcomes, such as tubular atrophy and fibrosis. In contrast, transitioning to OXPHOS metabolism has the potential to decelerate AKI progression and assist in the recovery of renal function.

While evidence underscores the crucial role of OXPHOS in kidney repair, the research landscape regarding the timing and regulation of this metabolic shift is limited in scope. Specifically, determining the optimal timing to initiate this metabolic shift—whether it should occur in the hyperacute phase, the days following the onset of AKI, or during the chronic phase—remains largely unexplored. Furthermore, although resistance and tolerance mechanisms are postulated in existing studies, there is a dearth of solid empirical evidence to support these claims, especially in the context of SA-AKI. Consequently, this highlights the necessity for comprehensive research to elucidate their specific roles and interactions. Additionally, despite the extensive research on various types of acute kidney injury, focused studies on SA-AKI are notably lacking. Given the potential variations in pathogenesis across different AKI types, further investigation is essential to bridge this knowledge gap.

As of now, there are no clinically approved therapeutic agents designed to modulate metabolic reprogramming, and the pharmacological safety profiles of such interventions are yet to be determined, calling for broad-ranging research. Nonetheless, the profound impact of metabolic reprogramming in TECs on SA-AKI progression emphasizes its potential as a focal point for future research. Therefore, targeting metabolic changes in TECs shows promise as a strategy for both preventing and treating SA-AKI.
